# Genome Sequence of a Lytic Staphylococcus aureus Bacteriophage Isolated from Breast Milk

**DOI:** 10.1128/mra.00953-22

**Published:** 2022-11-23

**Authors:** Joshua J. Iszatt, Alexander N. Larcombe, Luke W. Garratt, Stephanie Trend, Stephen M. Stick, Patricia Agudelo-Romero, Anthony Kicic, WAERP, AREST CF

**Affiliations:** a Occupation, Environment and Safety, School of Population Health, Curtin University, Perth, Western Australia, Australia; b Wal-yan Respiratory Research Centre, Telethon Kids Institute, The University of Western Australia, Perth, Western Australia, Australia; c Telethon Kids Institute, The University of Western Australia, Perth, Western Australia, Australia; d Centre for Neuromuscular and Neurological Disorders, Perron Institute for Neurological and Translational Science, The University of Western Australia, Perth, Western Australia, Australia; e Department of Respiratory and Sleep Medicine, Perth Children’s Hospital, Perth, Western Australia, Australia; f Centre for Cell Therapy and Regenerative Medicine, School of Medicine and Pharmacology, The University of Western Australia and Harry Perkins Institute of Medical Research, Perth, Western Australia, Australia; g Australian Research Council Centre of Excellence in Plant Energy Biology, School of Molecular Sciences, The University of Western Australia, Perth, Western Australia, Australia; h European Virus Bioinformatics Center, Jena, Thuringia, Germany; DOE Joint Genome Institute

## Abstract

We identified a double-stranded DNA (dsDNA) bacteriophage appearing to belong to *Herelleviridae*, genus *Kayvirus*. The bacteriophage, Biyabeda-mokiny 1, was isolated from breast milk using a clinical isolate of Staphylococcus aureus. The genome is 141,091 bp in length and encodes 230 putative coding sequences.

## ANNOUNCEMENT

Antimicrobial resistance (AMR) in bacteria is a growing concern for public health worldwide (1). One pathogen, Staphylococcus aureus (2), is known for its high rate of transient and persistent colonisation and its ability to develop resistance (3, 4). This has led to a resurgence of interest in bacteriophage therapy (BT) (5–7) and its therapeutic application (8, 9). Here, we report the genome sequence of Biyabeda-mokiny 1, a bacteriophage isolated using a clinical isolate of methicillin-resistant Staphylococcus aureus (MRSA).

Biyabeda-mokiny 1 was isolated from breast milk samples obtained from the COMET study (University of Western Australia [UWA], Perth, Western Australia, Australia), enriched using a MRSA clinical isolate, and propagated within an orbital shaker at 50 rpm and 37°C for 24 h. Phage were then purified by three rounds of single-plaque isolation and propagation on agar plates using its host as recommended in reference 10. Purified bacteriophage DNA was extracted from high-titer (1 × 10^9^) lysates using the DNeasy blood and tissue kit (Qiagen) and sent to the Australian Genome Research Facility (AGRF) for library preparation (Nextera XT). Illumina whole-genome sequencing using the NovaSeq 6000 platform (NovaSeq Control Software v1.7.5) was used to generate 3,690,362 150-bp paired-end (PE) reads for assembly. Adaptor trimming was done using Trimmomatic (v0.39) (11), and BBtools (v38.96) (12) was used to perform digital normalization to 500× and a minimum depth per read of 5×. Reads were then merged and assembled, *de novo*, using SPAdes Genome Assembly Algorithm (v3.15.4) (13). Draft assemblies were improved using Pilon (v1.24) with–drags parameter (14), and the polished assembly was then used to calculate depth coverage with both deduplicated and normalized reads using BBmap (12) to obtain the maximum and target coverage metrics. Annotations were performed using Prokka (v1.14.6) (15) in combination with the PHROG database (16). All tools were run with default parameters unless otherwise specified.

In addition, Basic Local Alignment Search Tools (BLASTn) (17) using the NCBI database (nucleotide) was used to obtain the genomes closest to Biyabeda-mokiny 1, and FastANI (v1.33) (18) was used for pairwise genome comparison by generating the average nucleotide identity (ANI) percentage (Table 1). Basic statistics such as length and GC content were calculated using Quast (v5.0.2) (19), and completeness was confirmed via CheckV (v0.9.0), identifying direct terminal repeats within the viral contig (20) (Table 1). All databases and software used were accessed on 29 July 2022 unless otherwise specified.

**TABLE 1:** Genome characteristics and comparative analysis of Biyabeda-mokiny 1 and its three closest relatives

Genome	Genome size (bp)	GC content (%)	Coverage (×)		No. of coding sequences	GenBank accession no. of closest relative	Alignment (% ANI)
			Deduplicated reads	Normalized reads			
Biyabeda-mokiny 1	141,091	30.44	1,730	588	220	JX878671.1	96.9967
						AP018375.1	97.6185
						MH107769.1	96.7908

Based on the genomic features previously described, and the pairwise alignment scores in Table 1, Biyabeda-mokiny 1 is likely to belong to *Herelleviridae*, particularly the genus *Kayvirus*. Similarly classed bacteriophages have been previously isolated, characterized, and identified as therapeutic candidates (21–24). These criteria have been based on previously published taxonomic guidelines (25).

All samples were collected under approval from relevant ethics committees. We declare that they have no conflicts of interest. This article does not contain any studies involving animals performed by any author.

### Data availability.

The assembled and annotated genome is available through GenBank under accession number OP263967, BioProject number PRJNA862986, and BioSample accession number SAMN30202906 (Biyabeda-mokiny 1), as well as Sequence Read Archive number SRX17131995. All scripts used for the assembly and analysis of raw reads are contained within a git repository publicly available at https://github.com/JoshuaIszatt/Phanatic.
